# Comparison of an integrated versus stand-alone approach to post-validation surveillance for lymphatic filariasis in Niue: a micro-costing study, 2025

**DOI:** 10.7189/jogh.16.04157

**Published:** 2026-05-08

**Authors:** Adam T Craig, Harriet LS Lawford, Grizelda Mokoia, Patricia Tatui, Misiona Nicolas, Amanda Murphy, Tonia Marquardt, Son Hong Nghiem, Anderson E Stanciole, Colleen L Lau

**Affiliations:** 1 Frazer Institute, The University of Queensland, Brisbane, Queensland, Australia; 2 Department of Health, Government of Niue, Alofi, Niue; 3 Australian Institute of Tropical Health and Medicine, James Cook University, Cairns, Queensland, Australia; 4 National Centre for Immunisation Research and Surveillance, Sydney, New South Wales, Australia; 5 Centre for Health Service Research, The University of Queensland, Brisbane, Queensland, Australia; 6 Global Institute for Disease Elimination, Abu Dhabi, United Arab Emirates

## Abstract

**Background:**

Lymphatic filariasis (LF) is a mosquito-borne parasitic disease which, in cases of chronic infection, may result in lymphoedema, elephantiasis, and hydrocele, imposing significant physical, social, and economic burdens. Global initiatives have reduced the prevalence of this condition, with Niue, a self-governing Pacific Island country, being validated as having eliminated LF as a public-health problem in 2016. To ensure transmission does not re-emerge, the World Health Organization recommends post-validation surveillance. As an integrated approach where LF-PVS is embedded within existing health programmes may reduce financial and labour costs and hence improve feasibility for resource constrained settings, we compare the costs of integrated LF-PVS in Niue with the counterfactual of stand-alone LF-PVS.

**Methods:**

We itemised activities and resources and assigned unit costs using a bottom-up micro-costing approach, drawing data from project budgets, accounting records, and staff reports. For the stand-alone scenario, we base LF survey inputs and activities on a recently implemented LF-PVS in a comparable context. For the integrated scenario, we embedded LF-PVS activities within a national STEPwise survey for non-communicable disease risk factors, with two research staff and one Department of Health officer responsible for implementation. All costs are reported in USD 2025 prices.

**Results:**

Total provider costs under the integrated scenario were USD 59 488 compared with USD 105 414 for the stand-alone. The integrated cost per participant tested was USD 59 compared to USD 105 for the stand-alone approach, representing a 44% reduction. The required Department of Health staff time was half in the integrated scenario, resulting in savings of approximately four weeks full-time equivalent staff time.

**Conclusions:**

This analysis provides evidence that programmatic integration of LF-PVS reduces cost to donors and lessens burden on public health staff. Future LF-PVS in the Pacific and elsewhere should consider integrating with routine health surveys to maximise operational efficiency.

Lymphatic filariasis (LF) is a neglected tropical disease caused by infection with thread-like filarial worms (*Wuchereria bancrofti*, *Brugia malayi*, and *B. timori*) [1]. Adult worms reside in lymphatic vessels for 6–8 years, producing, if mated, millions of microfilariae that circulate in the blood. These parasites are transmitted to humans through the bites of infected mosquitoes. While most infections are asymptomatic, chronic cases can lead to lymphoedema (tissue swelling due to lymphatic dysfunction), elephantiasis (thickening of the skin and underlying tissues) of the limbs, and scrotal hydrocoele [2]. These conditions can result in substantial physical impairment and are often associated with social stigma, which may lead to exclusion and loss of livelihood [3].

The Global Programme to Eliminate LF developed by the World Health Organization (WHO) has successfully reduced infection prevalence through mass drug administration (MDA), and 21 countries and territories, including Niue, have achieved the target of <1% microfilaria or <2% LF antigen prevalence required to declare that they have eliminated the disease as a public health problem [4,5]. Since 2000, 9.7 billion cumulative treatments have been delivered to over 943 million at-risk people through MDA [6]. Yet in 2023, the WHO estimated that 657 million people in 39 countries still live in areas requiring preventive chemotherapy [7,8].

Although activities of the Global Programme to Eliminate LF interrupted transmission in many countries, the risk of re-emergence persists. The WHO, therefore, recommends post-validation surveillance (PVS) after reaching elimination targets [5] be implemented to detect any residual or recrudescent transmission and to provide early warnings should prevalence begin to increase [9]. However, resource constraints, together with a shift in political and policy attention following the elimination of LF, have resulted in PVS not being implemented in many countries [10–12]. This highlights the need for cost-effective surveillance strategies suitable to epidemiological and contextual realities.

## Niue and previous lymphatic filariasis control activities

**Figure 1:**
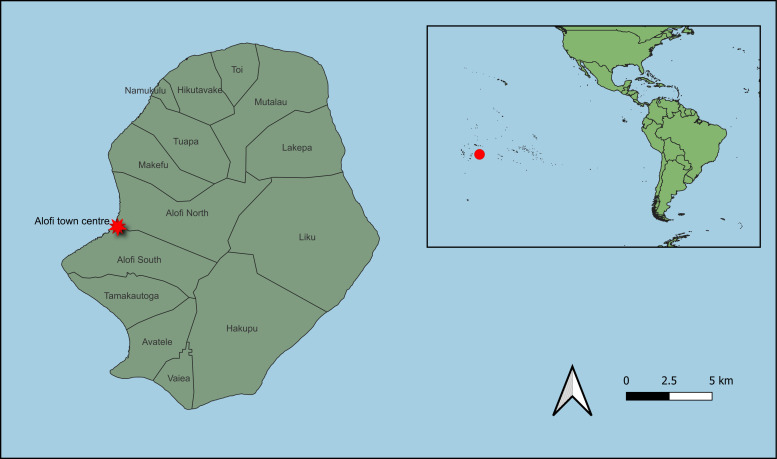
Map of Niue and its location in the South Pacific Ocean. The figure was produced using QGIS, version 3.40.8 – Bratislava (QGIS Foundation, Grüt, Switzerland). Base map data were sourced from the Pacific Data Hub [14] under a CC BY 4.0 license.

Niue is a self-governing island country in the South Pacific Ocean that covers roughly 261 km^2^ and has a resident population of around 1600 people [13] (**Figure 1**). The first LF survey in Niue was conducted in 1954 and found a population microfilariae prevalence of 22.1%. Subsequently, nine population-wide rounds of MDA supported by nine additional serosurveys health authorities successfully reduced the prevalence of LF antigen positivity to below the WHO’s threshold for elimination. The WHO validated Niue’s elimination of LF in 2016 [15–18]. Since then, surveillance for LF had not been conducted until this study.

Niue’s 2025–2026 national budget reflects a strong commitment to health as a core public service [19]. The country’s health system is supported by external funding, particularly from New Zealand and Australia, which contributes to infrastructure and operational activities, including specialised initiatives such as national surveys [19]. The Department of Health (DoH) operates with an annual budget of approximately USD 1.7 million, of which around USD 285 000 is allocated to public health [19].

In 2025, the Niue DoH, in collaboration with The University of Queensland (UQ) Operational Research and Decision Support for Infectious Diseases programme and their partners, implemented the country’s first LF-PVS [15], leveraging resources to embed LF-PVS activities within Niue’s planned WHO STEPwise survey for non-communicable disease risk factors [20]. This integrated approach sought to reduce costs and impost on a very small (~2.5 full-time equivalent) public health workforce by minimising the logistical and operational support required, while still collecting blood specimens and clinical data needed for LF PVS.

## Integrating surveys: evidence from other settings

The integration of disease surveillance activities into existing platforms has been encouraged to enhance efficiency. For example, a cost analysis in Burkina Faso compared stand-alone health and demographic surveillance with an integrated comprehensive disease assessment survey and found that fixed costs were similar, but variable costs were markedly lower in the integrated survey [21]. In this example, integrating activities saved approximately USD 45 000 per year and reduced the cost per household visit from USD 25 to USD 21.

Evidence on the costs and benefits of integration for PVS, however, remains sparse [22] and non-existent in small island developing state contexts. Therefore, we aimed to quantify the cost savings associated with integrating LF-PVS into the STEPwise survey in Niue and to identify where savings were made.

## METHODS

### Study design and perspective

We conducted a retrospective micro-costing analysis [23] comparing two scenarios: an integrated LF-PVS involving finger-prick blood collection and antigen testing using Alere filariasis test strip and confirmatory microscopy that was conducted alongside the national WHO STEPwise survey in 2025 [15,24], and the counterfactual of a stand-alone LF-PVS (*i.e.* PVS not conducted with any other activity). We evaluated costs from the perspective of the programme implementers (*i.e.* a UQ research team) and the national health system (*i.e.* the Niue DoH), without including household costs (*e.g.* time lost by participants) and societal costs (*e.g.* work absenteeism) in the analysis.

In the integrated approach, the reported costs represent only the incremental expenditure directly attributable to LF-PVS activities undertaken within the existing STEPwise platform, rather than the total combined cost of both surveys. Core STEPwise operations, such as community engagement, logistics, and data management, were funded and, therefore, excluded, except where additional resources were required to implement LF-PVS-specific tasks.

### Data sources and assumptions

We extracted cost data from budgets, invoices, and time-and-motion logs compiled during the 2025 integrated survey in Niue [24]. For the stand-alone scenario, the quantity and costs of inputs were derived from the UQ team’s recent experience implementing LF-PVS in other Pacific Island countries [25–27] and understanding of what would have been required if the LF survey had been conducted separately. All monetary values are presented in USD at 2025 prices. Key assumptions, based on authors’ field experience, cost schedules, and experience, included:

- Survey duration: both scenarios assume five weeks of field work (25 field days) covering all 14 villages in Niue.
- Personnel: for the stand-alone survey, three visiting researchers were required for data collection, while two visiting researchers and one DoH staff member were sufficient for the integrated survey, owing to shared tasks with the STEPwise team. In each scenario, international staff rotated after three weeks in the field.
- Travel: international airfares and local transportation were budgeted at 2025 rates.
- Accommodation and per diems: hotel accommodation was budgeted at USD 132 per person per night for visits of up to one week. For more extended stays, USD 495 per week was allowed for renting multi-room accommodation to house the visiting team. In line with the 2025 UQ schedule, per diem allowances were USD 99 per person per day for visiting staff. A gratuity of USD 33 per day was included for local staff members in both scenarios.
- Data collection, laboratory testing, and data analysis: diagnostic methods (filariasis test strip and confirmatory microscopy), the number of samples processed, and statistical analysis workflows were the same in both scenarios. Costs related to multiplex bead assay (MBA) analysis were excluded, as this method is not yet standard for LF-PVS. If used, this analysis would represent a fixed, rather than variable cost (*i.e.* identical across both scenarios).
- Community engagement and dissemination: in the integrated scenario, community engagement and dissemination activities (village meetings, radio shows, social media messaging) were conducted with the STEPwise survey. Because these activities and related logistics were integrated into existing STEPwise activities, they were not costed separately unless they generated additional expenditure. This approach avoids double counting and ensures that the integrated scenario reflects only incremental LF-specific costs. In the stand-alone scenario, separate engagements (and associated costs) were assumed.
- The number of survey participants was set to 1000 for both scenarios.

### Costing approach

We estimated costs by six PVS component activities: pre-survey preparation and study design; pre-survey community engagement; field-based survey implementation; laboratory work; statistical analysis; results dissemination. We estimated the quantity of resources used for each activity in both scenarios, multiplying it by unit costs and summed to generate total costs. We calculated integrated cost savings for DoH staff time as the difference in the number of DoH person-days between scenarios multiplied by the daily wage (**Online Supplementary Document**).

### Outcome measures

The primary outcomes were the total provider cost and the cost per person recruited in each scenario. We also calculated the incremental cost difference (stand-alone minus integrated) and the ratio of stand-alone to integrated expenses. Secondary outcomes included DoH staff days saved and their monetary value.

## RESULTS

### Overview of costs

The integrated survey cost USD 59 488, whereas the stand-alone survey was estimated to cost USD105 414, amounting to a difference of USD 45 926. The cost per person recruited and tested in the LF-PVS was USD 59 for the integrated survey compared with USD 105 for the stand-alone scenario, equating to a 44% reduction in cost per participant. The stand-alone scenario, therefore, costs approximately 1.7 times more to implement than the integration approach (**Online Supplementary Document**).

### Drivers of cost savings

#### Travel, accommodation, and per diems

The most considerable savings stemmed from reduced international travel and accommodation costs associated with the stand-alone approach. By deploying two, rather than three external staff and one instead of three DoH staff, the integrated survey required 35 fewer person-nights of accommodation and associated costs (*e.g.* per diem). In total, accommodation and per diem costs were USD 9768 under the integrated survey scenario compared with USD 15 642 under the stand-alone scenario. Airfare and local transport costs were USD 5841 in the integrated scenario and USD 13 946 in the stand-alone scenario. Thus, the integrated scenario resulted in USD 13 979 in saving associated with travel and accommodation for international staff.

#### Pre-survey community engagement and dissemination

Under the integrated approach, community engagement activities (village meetings, radio shows, and social media messaging) were undertaken as part of the STEPwise survey and thus required negligible additional costs (around USD 800) while stand-alone engagement was estimated to cost USD 9500 and required significant additional UQ and DoH staff time. Similarly, disseminating findings to the community (through public meetings, radio, and social media) when shared with the STEPwise survey in the integrated scenario incurs a relatively small financial cost (USD 792), whereas dissemination in the stand-alone scenario was costed at USD 9200.

#### Specimen collection and data management

Because STEPwise participants provided finger-prick blood for glucose and cholesterol testing, the additional 300*μ*L blood collected from the same puncture added minimal time and cost, whereas a stand-alone LF survey required dedicated blood collection, costing an estimated USD 23 100. Consent procedures and interview times were shared, resulting in further savings. While not captured in this analysis, the finger-prick blood collection process performed by the UQ team in the integrated scenario provided labour (and hence, cost) savings for the STEPwise survey.

#### Laboratory analysis and MBA testing

Field laboratory costs were similar in both scenarios (USD 15 906 for the integrated scenario *vs*. USD 22 704 for the stand-alone scenario), as the specimen numbers and test procedures remained unchanged. Statistical analysis costs were identical, at USD 2112 in both scenarios.

#### Impact on the Niue health workforce

Integration reduced the total number of DoH person-days from 38.1 to 20.1, resulting in a savings of approximately four weeks of full-time staff equivalent. Beyond the direct labour cost savings of USD 4752, the reduction in DoH staff time required under the integrated approach freed up opportunities for alternative public health activities.

## DISCUSSION

This micro-costing analysis demonstrates that integrating LF-PVS into Niue’s national STEPwise survey yielded substantial cost savings compared with a stand-alone PVS survey. Provider costs were reduced by 44%, lowering the cost per person surveyed from USD 105 to USD 59. The main drivers of these savings were reduced travel and accommodation costs, as well as the ability to share cost of implementation with STEPwise survey. In addition, the integrated approach was associated with cost savings through reductions in DoH staff time, releasing the equivalent of approximately four full-time working weeks, thereby freeing up labour to perform other essential public health functions. These findings indicate that integration not only reduces financial expenditure, but also helps alleviate pressure on fragile domestic health systems. In small-island settings where human resources are limited, such opportunity-cost savings may yield greater marginal benefit than equivalent financial savings.

Explicitly distinguishing incremental from total programme costs is important for interpreting these findings. The integrated scenario reflects additional LF-specific expenses layered onto an existing platform, rather than total joint survey costs. Clarifying this counterfactual distinction ensures comparability with other integration analyses and aligns with Global Health Cost Consortium guidance on transparency in cost attribution [28]. The magnitude of savings should be interpreted considering the assumptions described above, particularly those related to staffing and allocation of shared costs. While a formal sensitivity analysis was not conducted, the main findings are likely to be robust to plausible variations in key parameters, including changes in input quantities or unit costs on the order of ±20%.

Reducing demands on an already overstretched public health workforce [29-31] generates benefits beyond direct labour savings. In resource-constrained settings such as Niue, where health personnel routinely manage multiple responsibilities, streamlining service delivery and administration can improve efficiency, reduce duplication, and lessen the burden on communities. In this study, integration halved the administrative workload for DoH staff, who would otherwise have had to organise and implement two separate community surveys, while participants were spared the inconvenience of multiple engagements. Such advantages highlight the potential for programmatic integration where target populations overlap and workflows for data and sample collection can be aligned. A potential additional benefit of integration is increased participation in both LF-PVS and STEPS surveys, as community members may perceive greater value, or because additional testing is offered without placing further demands on their time. As demonstrated by the Niue study, higher participation rates improve population coverage and enhances the statistical power of studies. For small island developing states and other resource-limited contexts, embedding LF PVS within existing survey platforms represents a practical strategy for sustaining surveillance in the post-elimination era. Ministries of health should therefore systematically review scheduled surveys (*e.g.* non-communicable disease risk factor surveys, demographic and health surveys) to identify opportunities that minimise duplication and maximise efficiency, with the choice of host survey shaped by timing, epidemiological context, programme priorities, available resources, the willingness of coordinators, and leaders’ ambitions. In Niue, the STEPwise survey was selected as its target a demographic (*i.e.* middle-aged individuals, males, and those with travel history to LF-endemic countries) others have found to be more likely to return a positive LF result [32–34]. Significantly, the models compared here represent only two possible approaches, and decisions will inevitably be influenced by factors beyond cost, including technical and logistical feasibility, funding availability, epidemiological priorities, and political will. Nonetheless, systematically costing and comparing PVS options provides critical insights for programme design and policy decisions, particularly when costs, benefits, and system-level implications are considered holistically. The straightforward micro-costing methodology applied here provides a pragmatic tool for generating such insights and tailoring PVS implementation to local needs and constraints.

The relatively high cost per test reported here reflects both the logistical realities of delivering public health programmes in Pacific Island settings, where travel, accommodation, and supply costs are considerable, as are the inherent challenges of surveillance for rare or eliminated diseases. By design, PVS aims to detect very low levels of transmission, which necessitates substantial investment. These costs should therefore be interpreted as a strategic investment in sustaining elimination. Forgoing PVS risks recrudescence and the potential need to re-initiate targeted or community-wide MDA, an intervention that entails far greater financial, societal, and reputational costs. From this perspective, periodic surveys, despite their expense, are a cost-effective safeguard to protect decades of elimination efforts. Identifying cost-efficient approaches to PVS, such as programmatic integration, is therefore both prudent and appropriate.

Beyond programmatic integration, additional efficiencies could be achieved through the adoption of novel multi-pathogen serological surveillance platforms, such as MBAs [35,36]. These assays allow for the simultaneous measurement of antibody responses to up to 90 pathogen biomarkers from a single dried blood spot [36]. Given that survey implementation and labour represented the largest cost components in this study, it is logical to maximise informational yield from each survey. Designing surveys to address multiple diseases (*e.g.* integrated NTDs, vaccine-preventable diseases and arboviral disease antibody monitoring using MBA) would shift the cost paradigm and thereby improve overall value for money. While MBA technology is new and more research is needed to better understand how to interpret its result, there is evidence from vaccine serosurveillance studies that it is useful to inform public health action [36–38].

This study benefited from detailed micro-costing and the use of locally sourced data to estimate resource requirements. Nevertheless, several limitations must be acknowledged. First, while drawing on costings from recent experience implementing PVS elsewhere, assumptions regarding the stand-alone survey scenario, particularly those related to personnel requirements and survey duration, may have led to an over- or underestimation of costs. Second, some categories, including the valuation of staff time and the use of facilities and equipment, were estimated and may not reflect actual provider costs. Third, household/societal costs, such as participants’ travel time or impost on the community, while potentially substantial, were not captured. Fourth, the analysis focused on implementers’ costs and did not account for potential opportunity costs associated with disease recrudescence if PVS were not conducted. Finally, findings from Niue may not be directly generalisable to larger or more complex settings, where integration could present different logistical, financial, and political challenges. Despite these limitations, this study provides novel, real-world evidence on the costs of integrated vs stand-alone approaches to PVS. The findings are particularly timely, aligning with newly released WHO guidelines on PVS [5] and emerging fiscal challenges confronting public health programmes globally. Taken together, the results highlight the potential of integration as a cost-efficient, context-appropriate strategy to sustain LF-PVS in the post-elimination era.

## CONCLUSIONS

Embedding LF-PVS within the national WHO STEPwise survey in Niue resulted in a reduction of total provider costs by approximately USD 46 200 and a decrease in the cost per person surveyed by 44%. Additional savings were realised by alleviating pressure on an already overstretched health system, freeing up scarce human resources, and sparing communities from the burden of participating in two separate surveys. This study provides real-world evidence that integrated approaches represent a financially efficient and operationally feasible strategy for sustaining LF PVS in small island states. Wider adoption of such models could support timely detection of any resurgence while ensuring prudent use of limited public health resources.

## Additional material


Online Supplementary Document
