# Supplementary material for: Comparison of an integrated versus stand-alone approach to post-validation surveillance for lymphatic filariasis in Niue: a micro-costing study, 2025
Source: J Glob Health. 2026 May 8;16:04157. doi: 10.7189/jogh.16.04157 (PMC13154341; doi:10.7189/jogh.16.04157)
Supplement: Online Supplementary Document [file jogh-16-04157-s001.pdf]

Supplement to: Craig AT, Lawford HLS, Mokoia G, Tatui P, Nicolas M, Murphy A, Marquardt T, Nghiem S, Stanciole AE, Lau CL. Comparison of an integrated versus stand-alone approach to post-validation surveillance for lymphatic filariasis in Niue: a micro-costing study, 2025. J Glob Health. 2026;16:04157.

**Survey component**	**Item**		**Units**	**Cost per unit (USD)**	**Units**		**Cost estimate (USD)**	
					**Integrated approach**	**Stand-alone approach**	**Integrated approach**	**Stand-alone approach**
1. Preparation	Study design, field preparation	Staff (International)	Days	$ 528.00	5	5	$ 2,640.00	$ 2,640.00
	In-country preparation	Staff (National)	Days	$ 264.00	1	6	$ 264.00	$ 1,584.00
2. Pre-survey community engagement	Community engagement	Staff (international)	Days	$ 528.00		7	-	$ 3,696.00
		Staff (National)	Days	$ 264.00	1	7	$ 132.00	$ 1,848.00
	Travel	International travel	Trips	$ 1,056.00	-	1	-	$ 1,056.00
	Accomodation	Hotel	Nights	$ 132.00	-	7	-	$ 924.00
	Per-diem	Allowance (international)	Days	$ 99.00	-	7	-	$ 693.00
		Allowance (national)	Days	$ 33.00	-	7	-	$ 231.00
	Transport (inc fuel)	Vehicle hire	Days	$ 46.20	-	7	-	$ 323.40
	Communication	Phone credit	Days	$ 9.90	-	7	-	$ 69.30
	Communication materials	Development	Lots	$ 528.00	1	1	$ 528.00	$ 528.00
		Printing/dissemination	Lots	$ 132.00	1	1	$ 132.00	$ 132.00
3. Survey implementation	Travel	International travel	Trips	$ 1,056.00	4	6	$ 4,224.00	$ 6,336.00
	Accomodation	Short term apartment rental	Weeks	$ 495.00	5	5	$ 2,475.00	$ 2,475.00
	Per-diem	Allowance (international)	Days	$ 92.40	70	105	$ 6,468.00	$ 9,702.00
		Allowance (national)	Days	$ 33.00	25	-	$ 825.00	-
	Training	Staff (Interntional)	Days	$ 528.00	1	3	$ 528.00	$ 1,584.00
		Venue hire	Days	$ 198.00	-	1	-	$ 198.00
	Informed consent	Staff (International)	Days	$ 528.00	-	1	-	$ 330.00
		Staff (MOH)	Days	$ 264.00	1	1	$ 165.00	$ 165.00
	Blood collection	Staff (international)	Days	$ 528.00	25	38	$ 13,200.00	$ 19,800.00
		Staff (MOH)	Days	$ 264.00	13	13	$ 3,300.00	$ 3,300.00
	Interview/data collection	Staff (international)	Days	$ 264.00	-	15	-	$ 3,850.00
		Staff (MOH)	Days	$ 264.00	15		$ 3,850.00	-
	Consumables	Consumables	Items	$ 2.11	1000	1000	$ 2,112.00	$ 2,112.00
		Filarial Test Strips	Items	$ 2.84	1000	1000	$ 2,838.00	$ 2,838.00
	Transport (inc fuel)	Vehicle hire	Days	$ 46.20	35	105	$ 1,617.00	$ 4,851.00
4. Field laboratory	Laboratory space	Venue hire	Units	$ 198.00	-	1	-	$ 198.00
	Sample preparation/testing	Staff (International)	Days	$ 528.00	25	38	$ 13,200.00	$ 19,800.00
		Staff (Domesitic)	Days	$ 264.00	5	5	$ 1,320.00	$ 1,320.00
		Consumables	Units	$ 1.39	1000	1000	$ 1,386.00	$ 1,386.00
5. Analysis	Mf slide reading	Staff	Days	$ 528.00	0.3	0.3	$ 132.00	$ 132.00
	Statistical analysis	Staff (International)	Days	$ 528.00	4	4	$ 2,112.00	$ 2,112.00
6. Dissemination	Dissemination to public	Staff (international)	Days	$ 528.00	-	7	-	$ 3,696.00
		Staff (Domestic)	Days	$ 264.00	1	7	$ 132.00	$ 1,848.00
		Comms development	Lots	$ 528.00	1	1	$ 528.00	$ 528.00
		Priniting/dissemination	Lots	$ 132.00	1	1	$ 132.00	$ 132.00
		Travel	Trips	$ 1,056.00	-	1	-	$ 1,056.00
		Accomodation	Days	$ 132.00	-	7	-	$ 924.00
		Per diem	Days	$ 99.00	-	7	-	$ 693.00
		Transport (inc fuel)	Days	$ 46.20	-	7	-	$ 323.40
**IMPLEMENTATION**							$ 64,240.00	$ 105,414.10
MOH labour saved through integration (days)				$ 264.00	-18		-$ 4,752.00	
**TOTAL COSTS**							**$ 59,488.00**	**$ 105,414.10**
